# EBV latent membrane proteins promote hybrid epithelial-mesenchymal and extreme mesenchymal states of nasopharyngeal carcinoma cells for tumorigenicity

**DOI:** 10.1371/journal.ppat.1009873

**Published:** 2021-08-18

**Authors:** Nannan Zhu, Xiaoting Xu, Yan Wang, Mu-Sheng Zeng, Yan Yuan

**Affiliations:** 1 State Key Laboratory of Oncology in South China, Sun Yat-sen University Cancer Center, Guangzhou, China; 2 Institute of Human Virology, Zhongshan School of Medicine, Sun Yat-sen University, Guangzhou, China; 3 Guanghua School of Stomatology, Guangdong Provincial Key Laboratory of Stomatology, Sun Yat-Sen University, Guangzhou, China; 4 Department of Basic and Translational Sciences, University of Pennsylvania School of Dental Medicine, Philadelphia, Pennsylvania, United States of America; National Cancer Institute, UNITED STATES

## Abstract

EBV-encoded LMPs are consistently detected in nasopharyngeal carcinoma (NPC). Recent evidence suggests potential roles of LMP1 and LMP2A in Epithelial-to-mesenchymal transition (EMT) process in NPC. EMT engages in the generation and maintenance of cancer stem cells (CSCs) and confers on cancer cells increased tumor-initiating and metastatic potential, and higher resistance to anticancer therapies. However, how LMP1 and LMP2A regulate the EMT process to generate cells with different EMT states and its implications for tumor progression remain unclear. Here we report that LMP1 and LMP2A promote EMT that drives NPC cells from the epithelial-like state (E) (CD104^+^, CD44^low^) to epithelial-mesenchymal hybrid (E/M) state (CD104^+^, CD44^high^). Furthermore, LMP2A possesses an additional function in stabilizing LMP1 and increasing the level of LMP1 in NPC cells. The elevated LMP1 further forces the EMT to generate extreme-mesenchymal (xM) state cells (CD104^-^, CD44^high^). To define the tumorigenic features of cancer stem cells at different states in the EMT spectrum, E, E/M and xM subpopulations were isolated and tested for tumorigenic capability in a tumor xenograft animal model. We found that the cells with E/M phenotypes possess the highest tumor initiating capacity. However, the xM subpopulation exhibits increased vasculogenic mimicry, a hallmark of metastatic cancers. Taken together, coordinated action of LMP1 and LMP2A generates an array of intermediate subpopulations in the EMT spectrum that are responsible for distinct tumorigenic features of NPC such as tumor-initiation, vasculogenesis, and metastasis.

## Author summary

Intratumoral heterogeneity, characterized by the existence of distinct cellular populations within tumor lesions, poses a significant challenge for the treatment of high-grade cancers. Using an EBV-associated nasopharyngeal carcinoma (NPC) model, we sought to elucidate how a virus or its oncoproteins promote the establishment of cancer stem cells that comprises heterogeneous subpopulations. We found that the coordinated action of EBV membrane proteins LMP1 and LMP2A generates heterogeneous subpopulations of cancer stem cells in nasopharyngeal carcinoma by activating the epithelial-to-mesenchymal transition (EMT). Furthermore, the contributions of the different subpopulations to NPC oncogenesis were investigated. Results showed that cells with an epithelial/mesenchymal hybrid state (E/M) possess the highest tumor initiating capacity; and a highly mesenchymal state (xM) subpopulation exhibits increased vasculogenic mimicry. These finding suggest that cancer stem cells residing at various EMT states are responsible for distinct tumorigenic features such as tumor-initiation, vasculogenesis, and metastasis.

## Introduction

Cancer stem cells (CSCs) are subpopulations of cancer cells that share similar properties with normal stem cells, including self-renewal and differentiation. CSCs are responsible for the initiation of new tumors, metastasis and therapy resistance [1]. Epithelial-mesenchymal transition (EMT) programs serve as a necessary path to gain CSC characteristics [2,3]. Therefore, EMT is a central driver of tumor malignancy and presents a promising therapeutic target for metastatic and recurrent tumors [1,3]. EMT is not a binary switch but exhibits many intermediate epithelial-mesenchymal phenotypic states arrayed along the epithelial (E) to mesenchymal (M) spectrum [3–6]. Recently it was reported that breast cancer stem cells with an intermediate hybrid E/M state exhibit the highest tumorigenicity while forcing such cells into a fully mesenchymal state result in a poorly tumorigenic cell population [7].

Nasopharyngeal carcinoma (NPC) is one of the most aggressive head and neck cancers, with a high incidence in Southern China and Southeast Asia [8,9]. Cancer stem cells exist in NPC tumors and cell lines. Cancer stem cell-like side population cells were identified in several NPC cell lines [10]. CD44^+^ cells were isolated from NPC cell lines and exhibited the characteristics of cancer stem cell or cancer progenitor cells such as pluripotent markers, self-renewal ability and chemo- and radiation-resistance [11,12]. Epstein-Barr virus (EBV) infection has been regarded as an essential step in the pathogenesis of NPC [8]. In NPC tumors, EBV is in type II latency, where three EBV-encoded proteins, Epstein–Barr nuclear antigen 1 (EBNA1), latent membrane protein 1 and 2 (LMP1, 2A, 2B), as well as two viral small RNAs (EBER1 and 2) are expressed. LMP1 and LMP2A expression are consistently detected across NPC tumors [13] and implicated as critical modulators in NPC pathogenesis and cancer stem cell formation. LMP1 promotes tumor cell invasion, metastasis and self-renewal [14–17]. LMP2A induces EMT and stem-like cell self-renewal in NPC, suggesting its roles in the initiation, metastasis and recurrence of NPC [18]. However, how LMP1 and LMP2A regulate different EMT states and their implications for tumor progression in NPC remain unknown. In the current study, we attempted to address these questions and investigate the functional roles of LMP1 and LMP2A in the EMT process. This study revealed that LMP1 and LMP2A coordinate to activate EMT and generate heterogeneous cancer stem cell subpopulations in NPC. Furthermore, various subpopulations in distinct epithelial-to-mesenchymal states exhibit different specific tumorigenic features, leading to high tumorigenic and metastatic NPC.

## Results

### LMP1/LMP2A increases cancer stem cell characteristics of NPC cells

**Fig 1:**
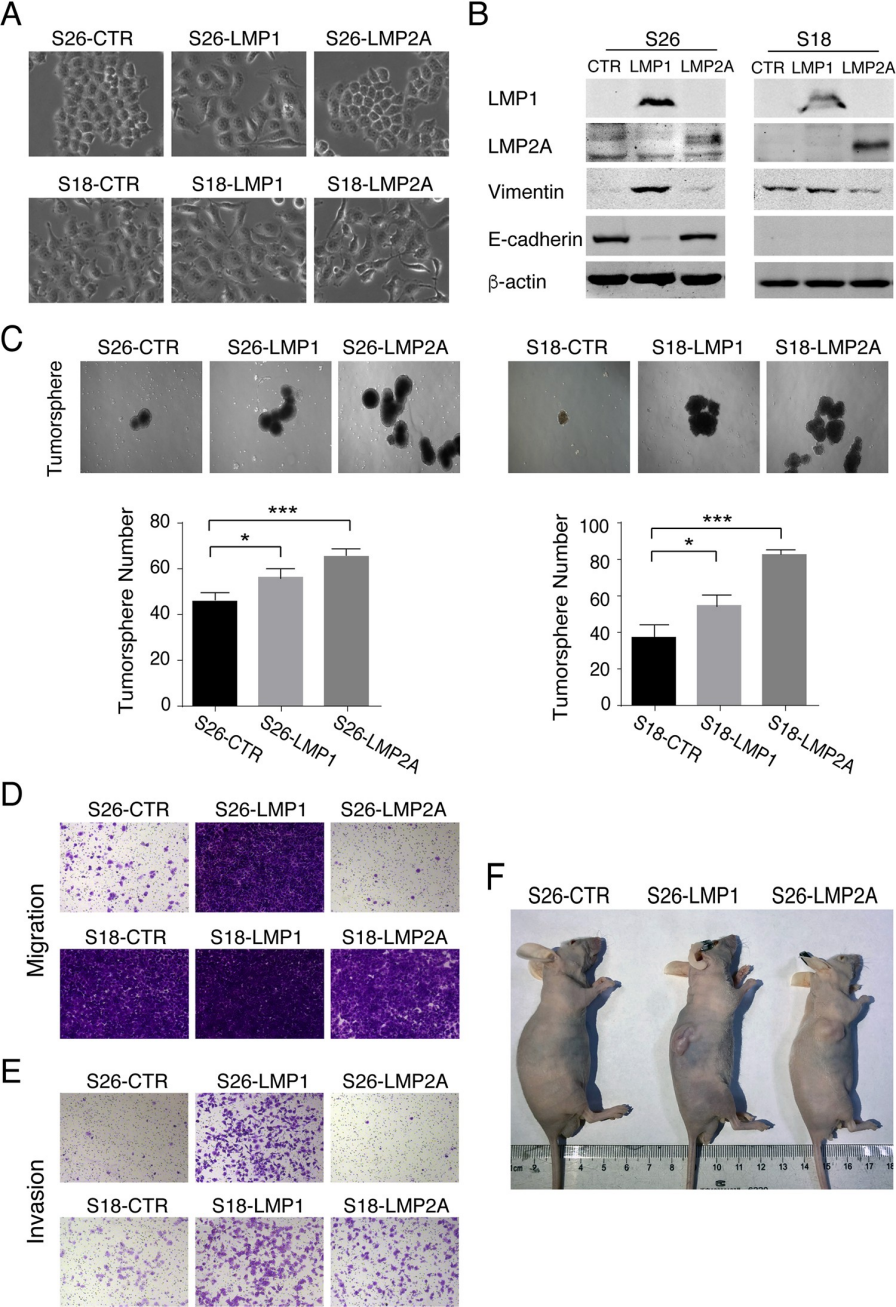
LMP1 and LMP2A enhance the CSC characteristics of NPC cells. **(A)** Morphological appearance of CNE-2 S26 and CNE-2 S18 cells and morphological changes of these cells in response to the expression of EBV latent membrane proteins LMP1 or LMP2A (200x). (**B)** Western blots of LMP1 and LMP2A, as well as EMT-associated markers Vimentin and E-cadherin. (**C)** Effects of LMP1 or LMP2A expression on tumorigenicity of S18 and S26 cells were assayed by tumorsphere formation assay. The numbers of tumorspheres of each sample were quantitated and representative images are shown (50x) (Mean+/- SD of three biological replicates, * P<0.05, *** P<0.0001). S18 and S26 cells and cells expressing LMP1 or LMP2A were assayed for their migration and invasion abilities using Transwell migration assay **(D)** and Transwell-Matrigel invasion assay **(E)**. Representative images are shown (100x). (**F)** S26 cells (S26-CTR) and S26 cells expressing LMP1 (S26-LMP1) or LMP2A (S26-LMP2A) were transplanted subcutaneously into the right flanks of BALB/C-nu/nu mice. The external views of tumor-bearing mice after 21 days are shown.

EBV latent membrane proteins LMP1 and LMP2A contribute to cancer stem cell generation [13,15,18]. To gain insights into the roles of LMP1 and LMP2A in CSC formation, we ectopically expressed LMP1 and LMP2A in an NPC cell culture model. S18 and S26 are two clones derived from the same NPC cell line CNE-2 but with different epithelial-mesenchymal phenotypic states. S18 displays mesenchymal-like (M-like) phenotypes and possesses great metastasis abilities, while S26 shows epithelial-like (E-like) phenotypes and produces low invasion and metastasis [19]. Morphologically, S26 are tightly attached, a typical epithelial phenotype (Fig 1A) due to the expression of E-cadherin (Fig 1B), while S18 cells lost epithelial phenotype including cell clustering (Fig 1A), suppression of E-cadherin, and up-regulation of Vimentin (Fig 1B). The expression of LMP1 in S26 cells resulted in a loss of cell-cell adhesion and polarity (Fig 1A), accompanied by increased mesenchymal phenotypes such as reduced expression of E-cadherin and increased expression of Vimentin (Fig 1B). In contrast, LMP2A had little effects on morphological features of S26. Neither LMP1 nor LMP2A changed phenotypes of S18, which already had mesenchymal phenotypes.

Then the effects of LMP1 and LMP2 on tumor initiation and cell invasion of NPC cells were analyzed. Tumorsphere forming assay is often used to study tumor initiation property of CSCs, and cell migration and invasion reflect tumor metastatic capability. Both LMP1 and LMP2 could significantly enhance tumorsphere formation, but LMP2A exhibited stronger capability in this CSC characteristic in S18 and S26 cells (Fig 1C). Furthermore, expressing LMP1 significantly increased the migration and invasion capacities of S26 cells and increased the invasion capacity of S18 cells (Fig 1D and 1E). When LMP1- and LMP2A-expressing S26 cells were implanted into nu/nu mice subcutaneously, tumors were observed in both LMP1 and LMP2A mice (Fig 1F). Taken together, our results indicate that both LMP1 and LMP2A contribute to the development of CSC characteristics in NPC cells.

### Characterization of intermediate epithelial-mesenchymal phenotypic states of NPC cells

**Fig 2:**
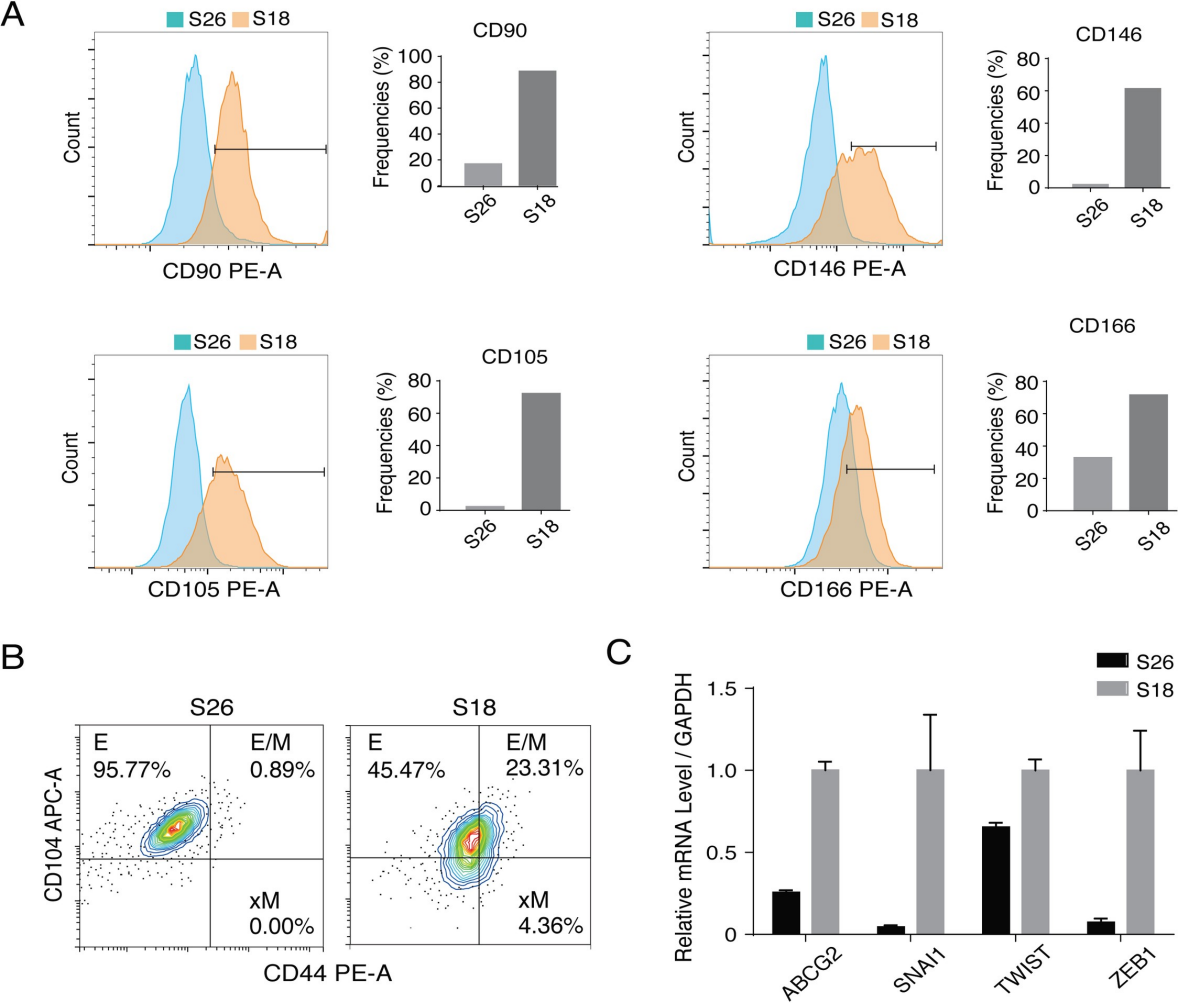
Epithelial-to-mesenchymal states of S18 and S26 clones of CNE-2 cells. (**A)** Flow cytometry histograms of EMT-associated markers CD90, CD146, CD105, and CD166 in S26 and S18 cells. (**B)** FACS profiles for CD44 and CD104 of S26 and S18 cells. (**C)** Relative mRNA expression level vs. GAPDH of EMT transcription factors (EMT-TFs) in S26 and S18 cells (Mean+/- SD of three biological replicates).

CSC characteristics are established through EMT. We hypothesized that LMP1 and LMP2A induce EMT to generate distinct epithelial-mesenchymal phenotypic states that correspond to different CSC characteristics. Using a series of mesenchymal and epithelial markers, we examined the epithelial-mesenchymal phenotypic states of NPC cells along the epithelial (E) to mesenchymal (M) spectrum. Flow cytometry analysis showed that S18 displayed high levels of mesenchymal markers CD90, CD146, CD105 and CD166, while S26 showed relatively low levels of these mesenchymal markers, confirming that S18 comprises a high proportion of cancer stem-like cells with mesenchymal features, while S26 cells are mainly in an epithelial state (Fig 2A).

Then, we analyzed the existence of distinct E-M states of S18 and S26 cells. The combination of CD104 and CD44 markers have been used in basal breast cancer cells to identify epithelial (E) (CD104^+^CD44^low^), Hybrid E/M (CD104^+^CD44^high^) and mesenchymal (xM) (CD104^-^CD44^high^) cell populations [7]. As expected, S26 cells were mainly in epithelial phenotype state, with more than 96% of cells expressing CD104^+^CD44^low^ (Fig 2B). However, S18 exhibited a mix of diverse tumor transition states of EMT, including subpopulation cells in E (45.5%), E/M (23.31%) and xM (4.36%) (Fig 2B). The expression of EMT transcription factors (EMT-TFs), including SNAI1, TWIST and ZEB1, were examined in S18 and S26 cells and results showed that the EMT-TF expression was elevated in S18 in comparison to S26 cells. The expression of SNAI1 is known to be up-regulated in the E/M cells; ZEB1 is a key regulator for activation of xM state in breast CSCs [7]. Therefore, the expression of EMT-TFs appears to be responsible for the maintenance of E/M and xM states of S18 cells (Fig 2C).

### LMP1 induces E/M and xM states and LMP2A promotes E/M state

**Fig 3:**
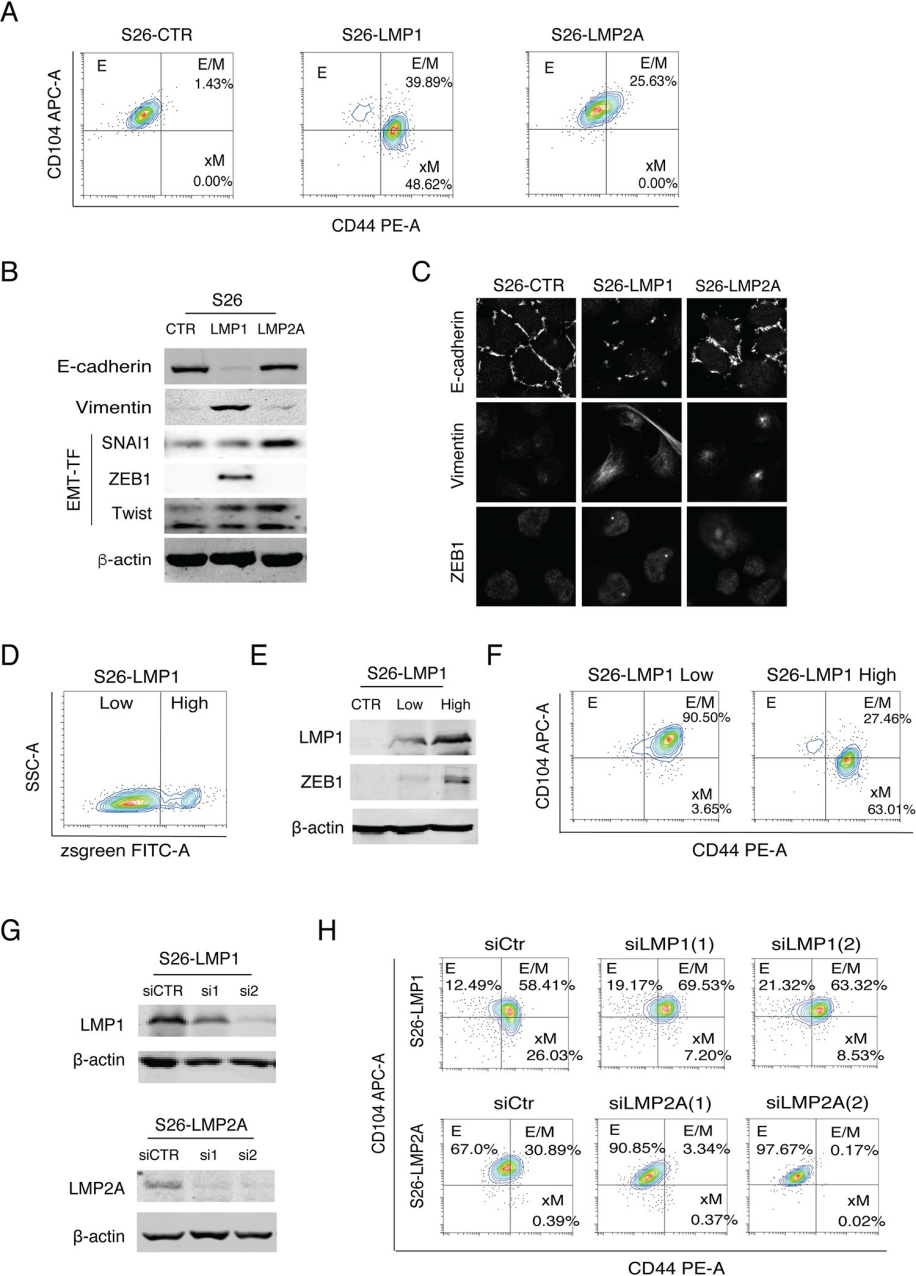
Effects of LMP1 and LMP2A expression on the generation of distinct subpopulations in the epithelial-mesenchymal spectrum. (**A)** The changes of CD44 and CD104 profiles of S26 cells in response to LMP1 and LMP2A expression revealed by flow cytometry analysis. (**B)** The expression of EMT markers (E-cadherin, Vimentin) and EMT-TFs (SNAI1, Twist, ZEB1) in S26-CTR, S26-LMP1, and S26-LMP2A cells were analyzed by Western blot. β-actin was included as a loading control. **(C)** Immunofluorescence assay of S26 cells (S26-CTR) and S26 cells expressing LMP1 (S26-LMP1) or LMP2A (S26-LMP2A) cells for the expression and subcellular localization of E-cadherin, Vimentin, and ZEB1 (630x). (**D)** S26 cells were transfected with a pLVX-EF1α-IRES-Zsgreen1-LMP1 plasmid that simultaneously expresses LMP1 and Zsgreen from a single mRNA transcript. LMP1^high^ and LMP1^low^ subpopulations were separated and isolated by FACS based on the fluorescence intensity of Zsgreen. (**E)** The LMP1 and ZEB1 expression levels in these two subpopulations were analyzed by Western blotting. (**F)** The epithelial-to-mesenchymal states of LMP1^high^ and LMP1^low^ subpopulations were determined on their CD44 and CD104 expression profiles. **(G)** S26-LMP1 and S26-LMP2A cells were transfected siRNAs to knock down the expression of LMP1 and LMP2A respectively. The knockdown efficiency of siRNAs was analyzed by Western blot. **(H)** The effects of LMP1 and LMP2A knockdown on CD44 and CD104 profile were analyzed by flow cytometry analysis.

To define the roles of LMP1 and LMP2A in EMT to generate distinct E to M transition states, we examined the changes in CD104/CD44 marker combination in response to LMP1 and LMP2A expression in S26 cells. The expression of LMP1 converted a large proportion of cells from E state to E/M (39.89%) and xM (48.62%) states. LMP2A expression drove 25.63% cells to the E/M state (Fig 3A).

It has been known that shifts between E and M states are governed by the action of EMT-TFs [20,21]. Knocking down the ZEB1 expression with shRNA resulted in failure to transit out of E state, and over-expression of ZEB1 forced cells entering into xM state in breast cells [7,22]. Therefore, we speculate that LMP1 and LMP2A promote EMT through activating certain signaling pathways leading to up-regulating EMT-TFs. The expression of EMT-TFs in response to LMP1 and LMP2A expression was analyzed. LMP1 significantly up-regulated the expression of ZEB1 revealed by both Western blot and IFA, accompanied by the loss of E-cadherin expression and the gain of mesenchymal marker Vimentin (Fig 3B and 3C). The expression of LMP2A did not up-regulate the expression of ZEB1 but elevated the levels of SNAI1 and Twist (Fig 3B). Given that SNAI1 is more important for orchestrating the E/M state, where ZEB1 is more important for the xM state [7], this result may explain why LMP2A only induces an E/M state, fails to achieve xM state.

Given that LMP1 induces ZEB1 expression and ZEB1 regulates the transition from E/M state to xM state, we wondered if the expression level of LMP1 in a cell controls the E/M to xM transition. To this end, we isolated LMP1^high^ and LMP1^low^ subpopulations from S26-LMP1 cells using FACS based on the fluorescence intensity of Zsgreen that were simultaneously co-expressed with LMP1 from a single mRNA transcript and served as an indicator of LMP1 expression (Fig 3D). The LMP1 expression levels in these two subpopulations were confirmed by Western blot (Fig 3E) and the E-M phenotypical states were analyzed with CD104/CD44 marker profile. Indeed, the low LMP1 expressing cells mainly resided in E/M (CD104^+^CD44^high^) state (95.5%), while the majority of the high LMP1-expressing cells (63%) had been converted to an xM state (CD104^-^CD44^high^) (Fig 3F). Western analysis of these two subpopulations demonstrated the correlation between high expression of ZEB1 and xM state, suggesting that LMP1-enhanced ZEB1 expression plays a crucial role in xM state formation (Fig 3E). To further confirm if levels of LMP1 and LMP2A expression determine the phenotypes of E/M and xM states, siRNA-mediated knockdown of LMP1 and LMP2A was performed on LMP1- and LMP2A-expressing S26 cells (Fig 3G). The result showed that reduction of LMP1 expression in S26-LMP1 cells by siRNA resulted in reduced xM state cells from 26.03% to 7.20%, and reduction of LMP2A in S26-LMP2A cells caused a decrease in E/M cells from 30.89% to 3.34% (Fig 3G and 3H). This result confirmed that LMP2A plays a role in the switch of NPC cells from E state to E/M state, and high expression of LMP1 further forces cells from E/M to xM state.

The effects of LMP1 and LMP2A on E–M state transition were also examined in the HK-1 NPC cell line, a differentiated squamous carcinoma line [23]. Results showed that LMP1 expression converted a large proportion of cells from E state to E/M (14.45%). LMP2A expression drove 4.58% to the E/M state (Supporting Information, S1 Fig). However, LMP1 failed to induce the xM state, probably because HK1 is a well-differentiated cell line and in a stable epithelial phenotype state with poor plasticity.

### An additional contribution of LMP2A to EMT by stabilizing LMP1

**Fig 4:**
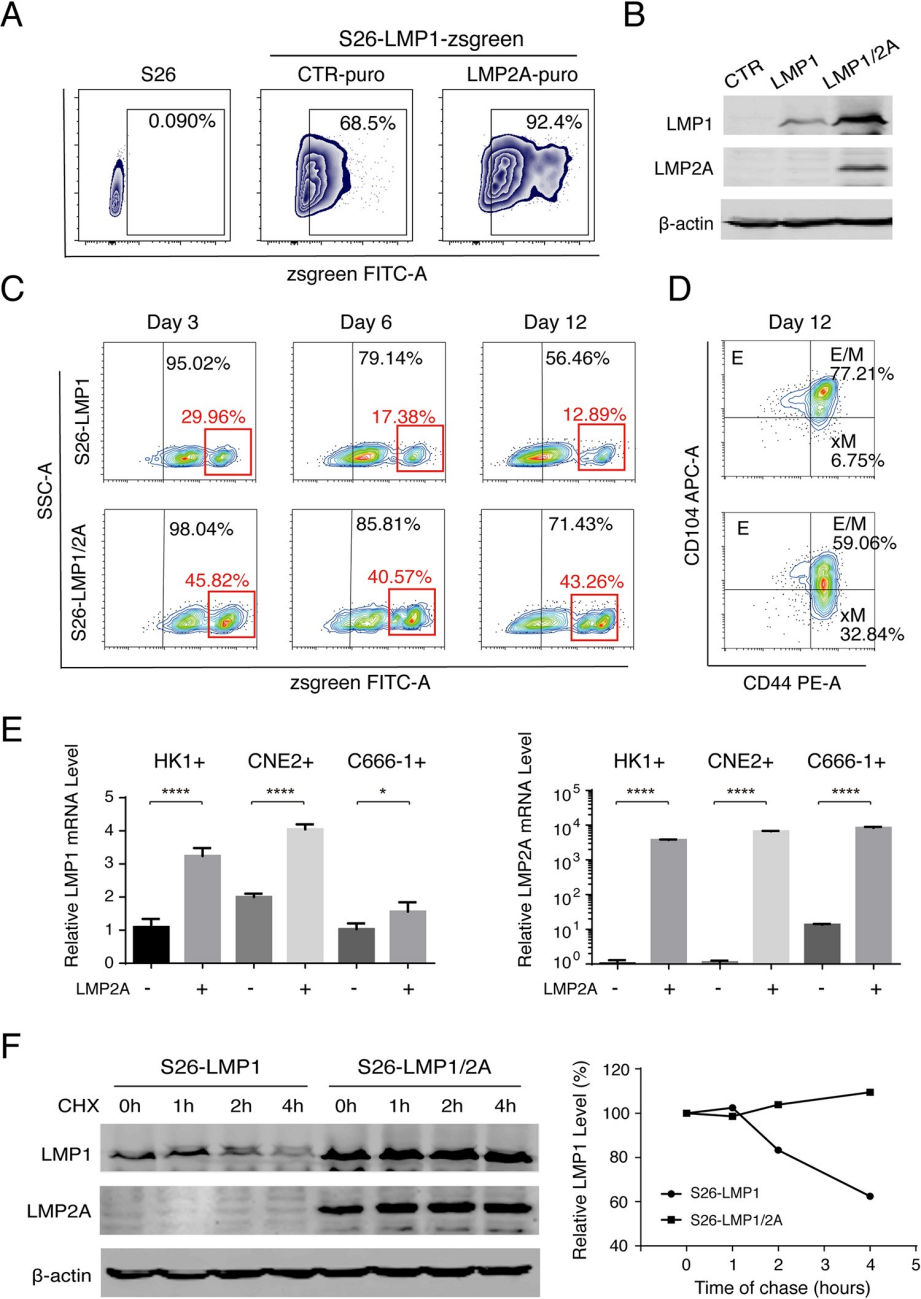
LMP2A contributes to EMT by stabilizing the LMP1 level in cells. **(A)** S26-LMP1-zsgreen stable cells were transduced with pLVX-EF1α-LMP2A-N1-Puro (LMP2A-puro) or CTR-puro lentiviral vectors (CTR-puro) to establish the S26-LMP1^zsgreen^/2A^puro^ (S26-LMP1/2A) and its control cell lines (S26-LMP1). The population of LMP1 positive (zsgreen positive) was analyzed by flow cytometry analysis. (**B)** The expression levels of LMP1 and LMP2A in S26-CTR, S26-LMP1 and S26-LMP1/2A cells were confirmed using Western analysis. (**C)** The maintenance of LMP1^high^ population (zsgreen^high^) in S26-LMP1 and S26-LMP1/2A cells over time after sorting (up to 12 days) was determined by flow cytometry. (**D)** The CD44 and CD104 FACS profiles of S26-LMP1 and S26-LMP1/2A cells were analyzed on Day 12. (**E)** Three EBV positive NPC cells (HK-1+, CNE2+, C666-1) were transduced with LMP2A expression lentiviral vectors or control (pLVX-EF1α-LMP2A-N1-Puro or CTR-puro). Relative mRNA expression levels (relative to GAPDH) of LMP1 and LMP2A in these cells are shown. (Mean+/- SD of three biological replicates). (F**)** S26-LMP1 cells and S26-LMP1/2A cells were treated with 100 μM cycloheximide (CHX) for 0, 1, 2, 4 hours, and the LMP1 and LMP2A levels were analyzed using Western blot and plotted against time. The data are represented as percentages of the protein detected at the beginning of the incubation (0 hours), all protein signals have normalized to β-actin.

LMP1 and LMP2A individually play roles in promoting EMT. Since both express simultaneously in NPC, a question was raised as to whether there is any synergy between LMP1 and LMP2A in regulating EMT. Towards this question, S26 cells were transfected with LMP1 gene alone and co-transfected with LMP1 and LMP2A, respectively. Co-transfection of LMP2A increased the expression level of LMP1 in S26 cells, revealed by flow cytometry (Fig 4A) and Western analysis (Fig 4B). Additionally, we sorted zsgreen positive cells from S26-LMP1 and S26-LMP1/2A by FACS and further cultured them for 12 days. The population of LMP1^high^ (zsgreen^high^) cells decreased with time when the cells expressed LMP1 alone. The percentage of zsgreen^high^ cells dropped from 29.96% on Day 3 to 12.89% on Day 12. However, when LMP1 and LMP2A were co-expressed in S26 cells, LMP1^high^ cells were maintained at high levels until Day 12 (Fig 4C). As a consequence, the co-expression of LMP1 and LMP2A resulted in an increased xM population from 6.75% to 32.84% (Fig 4D). To understand how LMP2A up-regulates LMP expression in NPC cells, we examined the effect of LMP2A on the transcription and protein stability of LMP1. CNE2-EBV+, HK1-EBV+, and C666-1-EBV+ cells were transfected with LMP2A. The LMP1 expression levels were significantly elevated in response to LMP2A expression in all three cell lines (Fig 4E), suggesting LMP2A expression facilitates LMP1 expression at the transcription level. Additionally, the LMP1 protein stability in cells in the presence or absence of LMP2A was also examined using the cycloheximide chase assay. As shown in Fig 4F, the stability of LMP1 dramatically increased when LMP2A was co-expressed in cells. Therefore, LMP2A up-regulates LMP1 expression in both transcription and protein stability levels. The contribution of LMP2A to EMT is two-fold: driving NPC cells to the E/M state and stabilizing LMP1 that promotes an xM state.

### E/M state cells exhibit the highest tumorigenicity and plasticity

**Fig 5:**
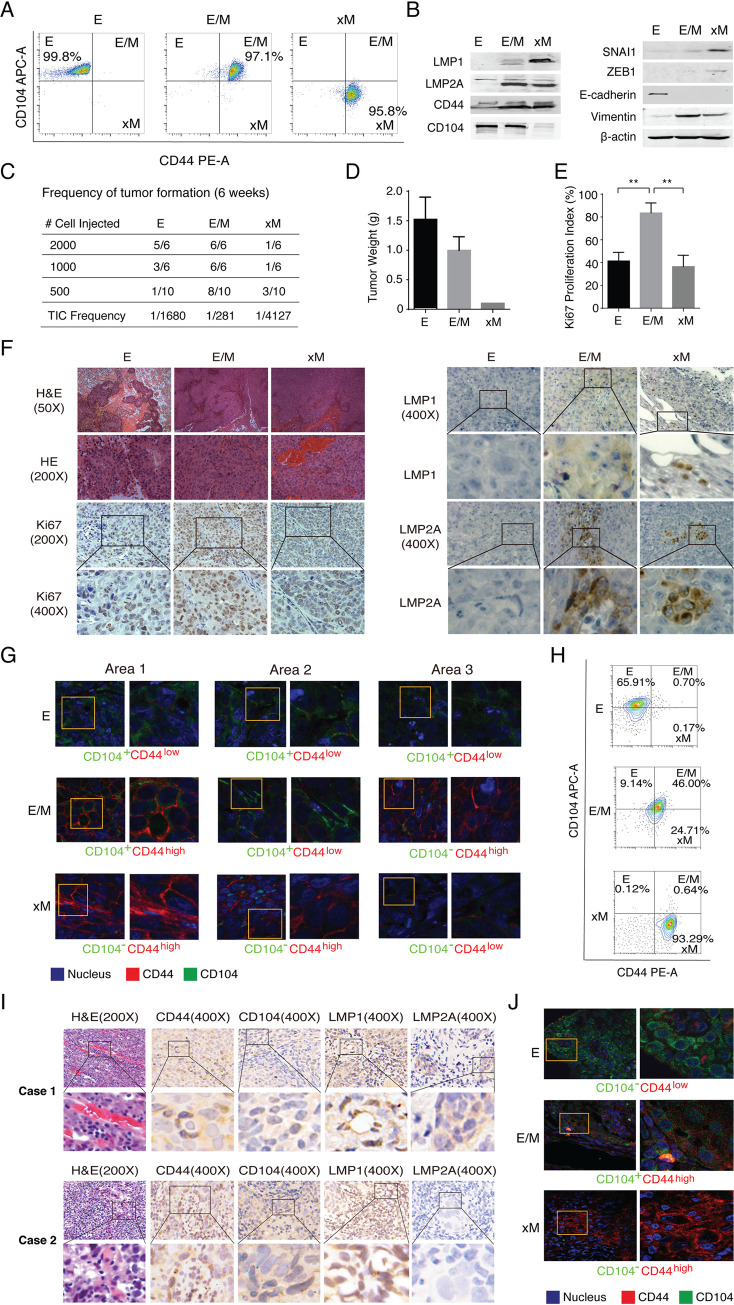
E/M state cells exhibit the highest tumorigenicity and plasticity. **(A)** E, E/M, and xM state cells were sorted from the S26 cells stably expressing LMP1 and LMP2A by FACS. **(B)** The expression levels of LMP1, LMP2A, CD104, CD44, EMT-TFs (SNAI1, ZEB1), and EMT markers (E-cadherin and Vimentin) in these subpopulations were determined by Western blot. (**C)** The sorted E, E/M, and xM cells were injected into BALB/c nude mice subcutaneously at limiting dilutions and the numbers of tumor formation were monitored weekly and counted after six weeks. Tumor initiation cell (TIC) frequencies (95% confidence intervals) were calculated using the extreme limiting dilution analysis (ELDA) [47]. (**D)** Then, mice were sacrificed and tumor weights were measured. **(E)** Tumors formed by E, E/M, and xM cells were analyzed by H&E staining, and the expression levels and localization of Ki67, LMP1, and LMP2A were analyzed by IHC assay. Ki67 proliferation index was assessed by point counting 500 cells and reported as the percentage of positive cells (Mean +/- SD of three randomly chosen fields under a microscope, 200x). **(F)** The representative images of IHC are shown. (**G)** CD44 (red) and CD104 (green) expression profiles in different areas of one tumor sample were analyzed by immunofluorescence assay (630x). (**H)** FACS profiles of CD44 and CD104 in cells isolated from tumors formed by E, E/M, and xM cells. **(I)** Three cases of NPC clinical samples were examined by H&E staining and the expression levels of CD44, CD104, LMP1, and LMP2A were detected by IHC assay. Two cases are shown. **(J)** Immunofluorescence assay to detect the localization and expression level of CD44 (red) and CD104 (green) in different areas of one NPC clinical sample.

To reveal the significance of distinct E–M states in NPC tumor initiation and progression, we isolated E, E/M and xM subpopulations from the S26 cells co-transfected with LMP1 and LMP2A by FACS. The cells of E, E/M, and xM states were confirmed to be CD104^+^CD44^low^, CD104^+^CD44^high^, and CD104^-^CD44^high^, respectively (Fig 5A). The expression levels of LMP1, LMP2A, CD104, CD44, and EMT-TFs in these subpopulations were determined by Western blot (Fig 5B). Cells of distinct subpopulations were injected into BALB/c nude mice subcutaneously at limiting dilution and the frequency of tumor-initiating cells (TIC) was determined for each subpopulation. Results showed that the hybrid E/M cells exhibited the highest TIC frequency (1/281), followed by E state cells (1/1680) and xM state cells (1/4127) (Fig 5C).

Then, the tumors formed by E, E/M and xM subpopulation cells at dilutions of 1000 cells per injection and 2000 cells per injection were weighted and analyzed by hematoxylin-eosin (H&E) staining. The tumors formed by E-state cells had a loose structure, even though they were heaviest in weight (Fig 5D and 5F). The tumors formed by E/M and xM cells were tightly structured with extravasated red blood cells (Fig 5F). The proliferative capacities of the tumors formed by different subpopulations were examined by immunohistochemistry (IHC) analysis for Ki67 expression, which is a marker of proliferative. Quantitative analysis of the immunohistochemistry images showed that the tumors formed by E/M cells exhibited the highest expression level of Ki67, suggesting that E/M state had a strong proliferative capacity (Fig 5E and 5F). IHC analyses for LMP1 and LMP2A were also carried out on these tumors and showed that only a fraction of cells in the tumors formed by E/M and xM subpopulations kept expressing LMP1 and LMP2A (Fig 5F).

The tumors formed by the E, E/M, and xM subpopulations were analyzed for CD44 and CD104 markers using immunofluorescence analysis. We found that the cells in the tumors formed by the E subpopulation were nonplastic, as shown by their invariable CD104^+^CD44^low^ phenotype. The tumors formed by E/M cells exhibited the highest plasticity *in vivo*, as E/M state cells (CD104^+^CD44^high^) could generate CD104^+^CD44^low^ and CD104^-^CD44^high^ phenotypes. Cells in xM tumors appear to be low plastic but a small number of cells were able to convert from CD104^-^CD44^high^ to CD104^-^CD44^low^, suggested that MET (mesenchymal-to-epithelial transition) had occurred in xM tumors in mice (Fig 5G). In addition, cells isolated from the tumors formed by E, E/M, and xM cells were analyzed by flow cytometry analysis for CD104 and CD44 profile and results confirmed that E/M state cells possess the highest plasticity, which converts to E state (9.14%) and xM state (24.71%). In contrast, 93.29% of the cells from the xM tumor maintain the xM state (Fig 5H). Overall, among distinct subpopulations of NPC CSCs, the E/M state possesses the highest tumor initiation ability and plasticity.

The plasticity of each subpopulation was further evaluated *in vitro* with purified E/M and xM state cells in which LMP1 and LMP2A expression was knocked down with specific siRNAs. Knockdown of either LMP1 or LMP2A could not revert xM phenotype to E/M, while silencing LMP1 or LMP2A resulted in reduced E/M state cells and increased E state cells (Supporting Information, S3 Fig), consistent with the *in vivo* observation above.

Three cases of NPC clinical samples were subjected to histochemistry assays, which allows a comparison of the tumors formed by E, E/M, and xM cells with NPC. H&E staining of these NPC samples showed abundant extravasation of red blood cells (two cases shown), similar to the tumors formed by E/M and xM cells (Fig 5I vs. Fig 5F). The expression of CD44, CD104, LMP1, and LMP2A in NPC clinical samples were analyzed by IHC assay and results showed that all three cases expressed LMP1, CD44, CD104, while only case 1 expressed a low level of LMP2A (Fig 5I). Furthermore, the NPC samples were examined for distinct E–M differentiation states by immunofluorescence analysis and results showed that the E (CD104^+^CD44^low^), E/M (CD104^-^CD44^high^) and xM (CD104^+^CD44^high^) prototypes existed in these NPC tumors (Fig 5J).

### xM state cells promote Vasculogenic mimicry

**Fig 6:**
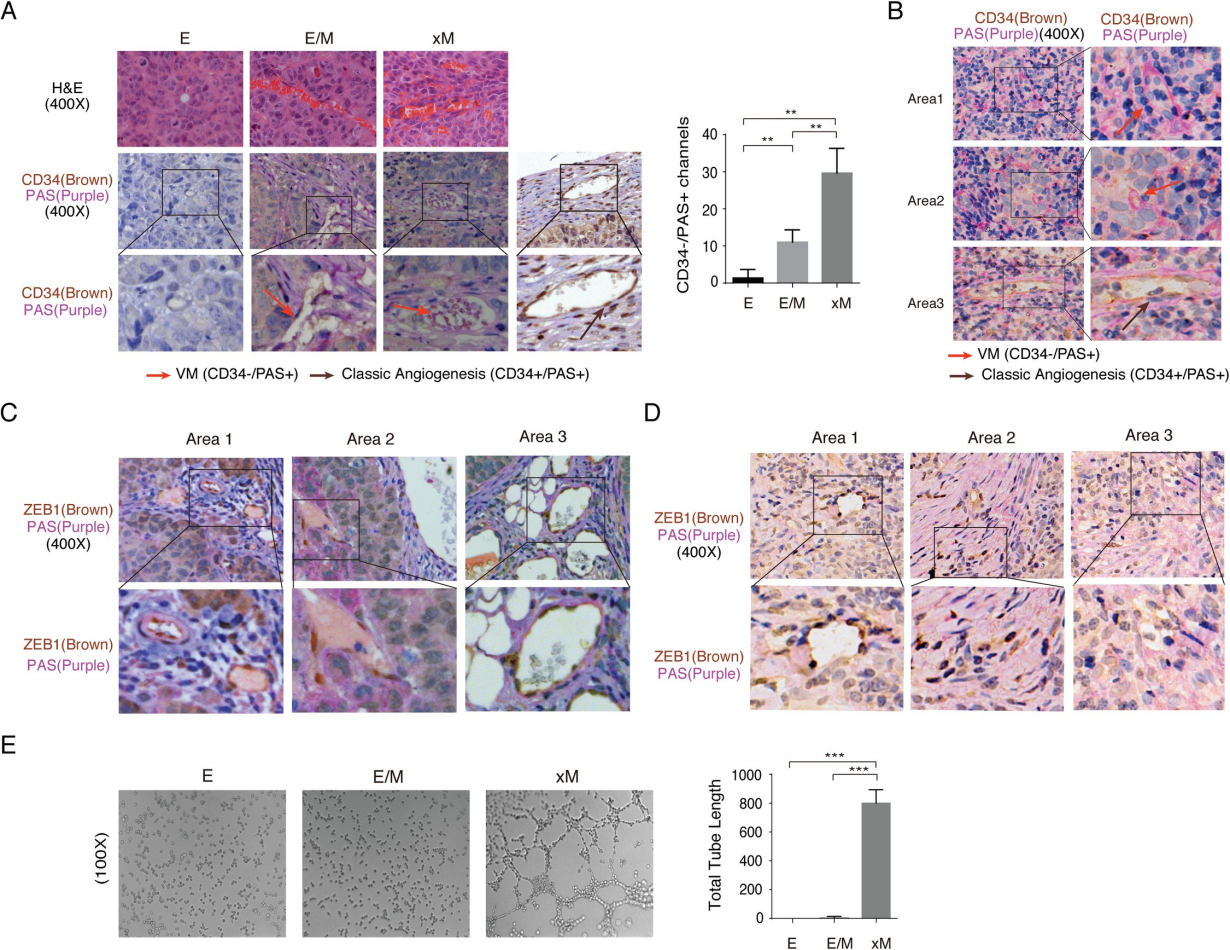
xM state cells are involved in vasculogenic mimicry (VM). **(A)** Tumors formed by E, E/M, and xM cells were analyzed by H&E staining and IHC analysis for the CD34 (Brown) and PAS (Purple). Pink arrows indicate VM channels (PAS+/CD34-), brown arrows indicate classic angiogenesis (PAS+/CD34+), which is included as a control. Quantification of VM channels determined by microscopy with 400x magnification in randomly chosen fields (n = 3 per group, ** P<0.01). (**B)** NPC clinical samples were analyzed by IHC analysis for detecting VM channels, three representative areas in one case are shown. (**C)** The colocalization of ZEB1 (Brown) and PAS (Purple) in xM tumor, as well as NPC clinical sample **(D)**. Images of three representative areas in one tumor sample are shown. **(E)** Vasculogenic mimicry of E, E/M, and xM cells was analyzed by Matrigel tube formation assay *in vitro* (Mean +/- SD of three biological replicates, *** P<0.001).

Although xM state cells showed low tumorigenicity and low plasticity, H&E staining of the tumors formed with xM cells showed abnormal and irregular vascular spaces with erythrocytes and abundant extravasation of red blood cells (Figs 5F and 6A). Neovascularity with extravasation of red blood cells is a histological feature of highly aggressive and metastatic tumors where blood vessels are formed by tumor cells instead of endothelial cells, called vasculogenic mimicry (VM) [24,25]. VM lacks an endothelial cell marker (CD34 or CD31) and has a basement membrane that stains positive with Periodic Acid-Schiff (PAS) regent [26,27]. It was recently reported that EBV can promote VM formation in NPC through activation of PI3K/AKT/mTOR/HIF-1a pathways [28,29]. The VM structures were defined as vascular-like channels positive for PAS but negative for CD34 and containing erythrocytes. To investigate the contribution of each subpopulation to vasculogenesis, tumors formed by E, E/M, and xM subpopulations were subjected to histochemical analysis of PAS and CD34. As shown in Fig 6A, tumors formed by xM state cells exhibited most VM structures, followed by E/M state cells. No VM was observed in tumors formed by E state cells. NPC clinical samples were also examined by histochemistry for PAS, CD34. VM was found in NPC samples as well (Fig 6B). ZEB1 is the key EMT-TFs for xM state cells. Double staining of PAS and ZEB1 were employed with tumors formed by xM cells to define the function of xM state cells in VMs formation. Results showed that the cells that participated in the formation of VM structures are ZEB1-positive (Fig 6C). The VM structures in NPC samples also exhibited ZEB1 positivity (Fig 6D).

Finally, we employed a 3D tube formation assay to test if xM exhibits the strongest VM formation ability *in vitro* among all subpopulations in the E–M spectrum. After seeding on the matrigel surface, only xM cells showed tubulogenic capacity in comparison with E and E/M state cells (Fig 6E). Thus, we conclude that the xM state possesses the highest capability of angiogenesis and VM formation both *in vitro* and *in vivo*. E/M state cells may obtain the capabilities of VM formation by trans-differentiated to xM state *in vivo* through highly expressing LMP1 and ZEB1.

## Discussion

Intratumoral heterogeneity, characterized by the existence of distinct cellular populations within a tumor lesion, poses a significant challenge for the treatment of high-grade cancers. This heterogeneity is thought to result from an epithelial-to-mesenchymal transition that generates an array of subpopulations of cells with various degrees of mesenchymal and cancer cell characteristics. These mesenchymal cancer cells, also called cancer stem cells, often exhibit increased tumor initiation capability, enhanced migration and metastasis, and elevated resistance to anticancer therapies. However, the detailed process of EMT and the biological significance for the existence of heterogeneous subpopulations have not yet been fully understood. In the current study, we attempted to elucidate how EBV oncogenic membrane proteins LMP1 and LMP2A drive EMT in nasopharyngeal carcinoma to generate heterogeneous cancer stem cell subpopulations. We found that LMP1 transforms E-like cells to obtain E/M and xM phenotypic states; while LMP2A only induces an E/M state. However, co-expression of LMP2A significantly elevates the level of LMP1, leading to increased xM state cells. We also investigated the roles of distinct CSC subpopulations in NPC tumorigenesis and found that the NPC cells with hybrid E/M state exhibit the strongest tumor initiation ability and plasticity. In contrast, xM states subpopulation contributes to vasculogenic mimicry in NPC.

Latent membrane proteins (LMPs) are EBV oncogenic proteins expressed in type II and type III latently infected cells. LMP1 is one of the EBV latent genes required for B cell immortalization and lymphoblastoid cell line (LCL) generation [30]. In non-lymphoid cells, LMP1 promotes transforming properties in fibroblast and epithelial cells, conferring anchorage-independent growth, enhanced motility/invasion, and forming tumors in nude mice [31–33]. LMPs are consistently detected across NPC tumors with variable levels of LMP1 protein with foci of positivity in the tumor [34,35]. This observation suggests the unique roles of LMPs in NPC development, which has not been fully understood. Recently, increasing evidence indicated that LMP1 and LMP2A may contribute to epithelial-mesenchymal-transition (EMT) and cancer stem cell formation in NPC [15,16,18]. LMP1 down-regulates E-cadherin through modulating EMT transcription regulators Twist and SNAI1, which is accompanied by a profound morphological change from cuboidal epithelial cells to elongated spindle-shaped cells, a characteristic of cells undergoing EMT [15,36,37]. These studies demonstrated that LMP1 and LMP2A contribute to NPC initiation, progression, and metastasis by generating and maintaining cancer stem cells in NPC via the EMT process. In this investigation, we initially found that both LMP1 and LMP2A can promote NPC cell conversion from the epithelial-like state (CD104^+^ CD44^low^) to epithelial-mesenchymal hybrid (E/M) state (CD104^+^ CD44^high^). This observation appears to show a redundant function of LMP1 and LMP2A in promoting EMT, consistent with previous reports that both LMP1 and LMP2A play roles in cancer stem-like cell formation and the expression of stem cell markers in cell lines and NPC biopsies [18,38,39]. Further investigation revealed an additional function of LMP2A in increasing LMP1 level in NPC mesenchymal cancer cells. The elevated LMP1 further forces the EMT to generate extreme-mesenchymal (xM) state cells (CD104^-^ CD44^high^). These results provide novel insights into the functions of LMP1 and LMP2A in EMT in NPC and pave the road to continue investigation into the mechanism of oncogenic virus EBV promoting cancer stem cell development.

LMP1 and LMP2A are integral membrane proteins. LMP1 functions as a constitutively active viral mimic of CD40 [35,40]. It was reported that CD40 ligation plays a role in regulating epithelial cell differentiation and inducing endothelial cell angiogenesis through activation of the PI3K pathway [41–43]. In this study, we showed that LMP1 activates EMT-TFs such as ZEB1 that is crucial for the acquisition of E/M and xM states in NPC. We also found that LMP1 activates mTOR signaling that is required for ZEB1 activation [44]. Taken together, we propose that LMP1 mimics CD40 ligation to induce E/M and xM state cells and increase vascular mimicry through activating ZEB1 via PI3K/mTOR pathway. LMP2A was reported to induce EMT and increases the number of cancer stem-like cells, which is correlated with the activation of the PI3K/AKT/mTOR pathway and up-regulation of EMT-TF SNAI1 [18]. Our result is consistent with the finding and showed that LMP2A enhances SNAI1 expression that renders NPC cells in the E/M state. In addition, LMP-mediated activation of the PI3K/AKT/mTOR pathway was also found to contribute to VM formation in EBV-associated epithelial malignancies [28]. Therefore, PI3K/AKT/mTOR signaling and the consequent up-regulation of EMT-TFs is possibly the central mechanism in LMP-mediated EMT that generates distinct subpopulations of CSC in NPC.

Multiple subpopulations of CSCs are present within a tumor, of which each may have unique biological characteristics [45]. The residence in the hybrid epithelial/mesenchymal (E/M) state has been reported to possess high tumorigenicity in breast cancer cells [7]. In NPC, LMP1 and LMP2A coordinately drive the EMT program, leading to the generation of an array of cell populations in the EMT spectrum (E, E/M, and xM) from a cloned NPC line. What is the biological significance of the existence of CSC subpopulations in a tumor? Intratumoral heterogeneity is associated with the grade of cancer malignancy. It is believed that heterogeneity leads to cancer adaptation to environmental and therapeutic pressure [46]. However, the mechanical details about the heterogeneous subpopulations in cancer development remain elusive. In the current study, NPC cancer stem cells at distinct states in the E-M spectrum induced by LMP1 and LMP2A were found to contribute to different oncogenic properties. The hybrid E/M cell population, defined by CD104^+^CD44^high^, had the highest tumor initiation ability and tumor formation in mice. In contrast, the subpopulation in the xM state defined by CD104^-^DC44^high^, showed poor tumor initiation ability but exhibited a higher angiogenesis capacity *in vitro* and displayed a strong ability to form vasculogenic mimicry (VM) *in vivo*. VM describes the process frequently observed in highly aggressive tumors to generate vascular-like structures or abnormal channels lined by highly malignant neoplastic cells without the participation of endothelial cells. VM plays critical roles not only in neoangiogenesis for tumor blood supply, but also in cancer progression dissemination and invasion for metastasis [25]. Our results suggest that NPC cells of xM state may be prone to participating in VM and therefore contribute to tumor metastasis. Further study to elucidate the mechanism underlying the establishment of particular CSC subpopulations and contribution of each subpopulation to tumor initiation and metastasis is warranted, which will suggest novel strategies to treat high-grade NPC by effectively targeting LMPs or specific CSC subpopulations.

## Materials and methods

### Ethic statements

The use of NPC clinical samples in the research were approved by the Sun Yat-sen University Cancer Center (Approval No. YB2016-076), in accordance to ICH GCP guidelines, government regulations, and laws. Written informed consent was provided by study participants. Animal experiments were approved by the IACUC of SYSU School of Medicine (No. 2017–196). Experiments were carried out under the institutional guidelines of caring laboratory animals, published by the Ministry of Science and Technology of People’s Republic of China.

### Cell culture

CNE2-S18 (S18), CNE2-S26 (S26), CNE2, and EBV-positive CNE2 cells were maintained in RPMI 1640 medium supplemented with 5% fetal bovine serum (FBS, Gibco Life Technologies). HK-1, EBV-positive HK-1, and C666-1 cells grew in RPMI medium with 10% FBS. HEK293T cells, purchased from American Type Culture Collection (ATCC), were cultured in Dulbecco’s Modified Eagle’s Medium (DMEM) supplemented with 10% FBS. All cultures contained 100 U/ml penicillin-streptomycin (HyClone).

### Antibodies

Antibodies against Vimentin (Cat#5741), SNAI1 (Cat #3879), ZEB1 (Cat #3396), SOX2 (Cat #3579), E-Cadherin (Cat #14472), TWIST1(Cat #46702), Ki67(Cat #9129) were purchased from Cell Signal Technology. Antibody against EBV LMP1 was purchased from Abcam (Cat #ab78113). Antibody against EBV LMP2A was purchased from Santa Cruz Biotechnology (Cat #sc-101314). CD44 PE (Cat #12-0441-81), CD104 (Cat #PA5-79541), CD104 eFluor 660 (Cat #50-1049-82) antibodies were purchased from ThermoFisher Scientific. CD44 antibody was purchased from Proteintech (Cat #15675-1-AP). Antibodies against CD90 (Cat #555596), CD146 (Cat #550315), CD105 (Cat #560839), CD166 Antibody (Cat #560903) were purchased from BD Bioscience.

### Plasmids

pLVX-EF1α-IRES-Zsgreen1 (Cat#VT2014) and pLVX-EF1α-Luc-N1 (Cat#VT9009) plasmids were purchased from Youbio, China. PSG5-LMP2A was provided by Musheng Zeng at Sun Yat-sen University Cancer Center. EBV LMP1 and LMP2A genes were cloned into the EcoRI and XbaI site of the pLVX-EF1α-IRES-Zsgreen1 and pLVX-EF1α-Luc-N1 vectors using ClonExpress II One Step Cloning Kit (Vazyme, Cat#C112-02). The PCR fragment of LMP1 was amplified from total DNAs of C666-1 cells using the following primers: 5’-GGATCTATTTCCGGTGAATTCATGGAACACGACCTTGAG-3’ (forward); 5’-CGCGGCCGCTCTAGATAAGAGTGCCATCTATCTGTACT-3’ (reverse). The PCR fragment of LMP2A was amplified from PSG5-LMP2A plasmid using the following primers:

5’-GGATCTATTTCCGGTGAATTCCAGAAATGGTGCCAAT-3’ (forward); 5’-CGCGGCCGCTCTAGACTACAAGCTAGCGTAATCT-3’ (reverse).

### Establishment of NPC cell lines stably expressing LMP1 and LMP2A

To produce LMP1 and LMP2A expression lentiviral particles, HEK293T cells were co-transfected with The pLVX-EF1α-IRES-Zsgreen1-LMP1, pLVX-EF1α-LMP2A-N1-Puro, and control vectors with packaging plasmids psPAX2, pMD2.G in a ratio of 4:3:1. 72-hour post-transfection, culture media were harvested and lentiviral particles were concentrated by ultracentrifugation. NPC cells were transduced with the lentiviruses in the presence of polybrene (8 μg/ml). The LMP1-Zsgreen or LMP2A-Zsgreen positive cells were sorted by FACS. The stable cell line S26-LMP1-Zsgreen were further transduced with LMP2A-puro lentiviruses to establish the S26-LMP1^Zsgreen^/2A^puro^ cell line (S26-LMP1/2A). S26-LMP1/2A cells were selected for 7 days with 2 μg/ml puromycin.

### Flow cytometry and cell sorting

Tumors were minced into 1 to 3 mm^2^ fragments and disaggregated using collagenase IV (6 mg/ml, Worthington Biochemical) and dispase (8mg/ml, Worthington Biochemical) for 1 hour at 37°C. The single cells were plated on 100 mm culture dishes with complete RPMI 1640 medium. After 48 hours, nonadherent cells were removed and the plastic-adherent cells were trypsinized for single cell suspension. 5x10^5^ cells (50 μl) were stained with appropriate diluted antibody for 20 min in room temperature and analyzed using BD LSRFortessa or CytoFLEX Flow Cytometer.

S26-LMP1^low^ and S26-LMP1^high^ populations were sorted according to the intensity of Zsgreen (FITC). E/M and xM populations were sorted from S26-LMP1/2A stable cells according to the expression level of CD44 (PE) and CD104 (APC).

### Cycloheximide chase assay

S26-LMP1 and S26-LMP1/2A cells were exposed to 100 μM cycloheximide (Beyotime, Cat#SC0353) for indicated hours, and Western blotting was performed to detect the proteins of interest.

### Western analysis

Cells were lysed in lysis buffer (50 mM Tris-HCl, pH 7.4, 150 mM NaCl, 1% NP-40, 1 mM sodium orthovanadate [Na3VO4], 20 mM sodium pyrophosphate, 100 mM sodium fluoride, 10% glycerol, protease inhibitor cocktail [1 tablet in 50 mL lysis buffer]). The cell lysates were homogenized and centrifuged at 13,000 rpm for 10 min at 4°C. The whole cell extracts of 50 μg protein was resolved by SDS-PAGE and transferred onto nitrocellulose membranes. The membranes were blocked in 5% non-fat milk in 1×PBS for 1 hour, and then incubated in diluted primary antibodies overnight at 4°C. IRDye 680LT and 800CW goat anti-rabbit IgG or anti-mouse IgG antibodies (LI-COR Biosciences) was used as secondary antibody. An Odyssey system (LI-COR) was used for detection of proteins of interest.

### RNA isolation and qRT-PCR analysis

Total RNA was extracted using Ultrapure RNA Kit (CWBIO, Cat#CW0581) according to the manufacturer’s instructions. Reverse transcription was performed to generate cDNA template. Relative mRNA expression levels were quantified by real time PCR on a Roche LightCycler 480 instrument and normalized to GAPDH. The primers used were as follows:

GAPDH: 5’-CATCATCCCTGCCTCTACTG-3’(forward) and 5’-GCCTGCTTCACCACCTTC-3’(reverse).
SNAI1: 5’-TCGGAAGCCTAACTACAGCGA-3’ (forward) and 5’-AGATGAGCATTGGCAGCGAG-3’ (reverse);
ZEB1: 5’-TTACACCTTTGCATACAGAACCC-3’ (forward) and 5’-TTTACGATTACACCCAGACTGC-3’ (reverse);
ABCG2: 5’-GGGTTCTCTTCTTCCTGACGACC-3’ (forward) and 5’-TGGTTGTGAGATTGACCAACAGACC-3’ (reverse).
TWIST1: 5’-GTCCGCAGTCTTACGAGGAG-3’ (forward) and 5’-GCTTGAGGGTCTGAATCTTGCT-3’ (reverse);
LMP1: 5’-CGTTATGAGTGACTGGACTGGA-3’ (forward) and 5’-TGAACAGCACAATTCCAAGG-3’ (reverse);
LMP2A: 5’-CGACCGTCACTCGGACTATCA-3’ (forward) and 5’-TTCCTCTGCCCGCTTCTTC-3’ (reverse);

### Tumorsphere formation assay

S18 and S26 cells, as well as the cells expressing LMP1 and LMP2A were plated at a density of 200 cells per well in Ultra-Low Attachment Multiple Well Plates (Corning, Cat#CLS3471). Tumorspheres were cultured for 10 days using serum-free DMEM/F12 medium supplemented with B27, 20 ng/ml epidermal growth factor and 10 ng/ml basic fibroblast growth factor. Spheres (>20 μm) were counted and visualized by a Zeiss observer Z1 microscope.

### Transwell migration and invasion assays

Cells, that had been starved for 24 hours, were resuspended at a final concentration of 1x10^6^ cells/ml in serum free RPMI 1640 medium. For migration assay, 100 μl of the cell suspensions was added into the upper chamber of Transwell insert (Corning, Cat#3422). Then 600 μl of RPMI 1640 medium with 20% FBS was added to the bottom well of the Transwell units. After 8 hours incubation at 37°C, cells that had passed through the filter were stained with crystal violet. The number of migrated cells was counted from multiple randomly selected microscopic visual fields using ImageJ software. Photographs were obtained and independent experiments were performed in triplicate. For invasion assay, the upper chamber of Transwell insert were coated with 50 μl of diluted (1:10) Matrigel and incubated at 37°C for 20 hours. Cells that have invaded into the lower chamber were fixed for 15 minutes with Ethanol and stained with crystal violet. The number of invaded cells were counted and visualized by Zeiss observer Z1.

### Immunofluorescence assay (IFA)

S26 cells and cells expressing LMP1 or LMP2A, were grown on glass coverslips (NEST) for 48 hours. Cells were fixed by using 4% paraformaldehyde for 10 min, permeabilized in 0.1% Triton X-100 for 30 min, and blocked in 1% BSA for 1 hour. Then the cells were incubated with anti-Vimentin, anti-ZEB1, or anti-E-cadherin antibodies for 2 hours at room temperature. Fluor Alexa-555 conjugated anti-rabbit IgG and Fluor Alexa-555 conjugated anti-mouse IgG was used as secondary antibody.

For immunofluorescent staining of paraffin-embedded tumors, sections were deparaffinized and rehydrated, followed by heat-mediated antigen retrieval. Slides were permeabilized in 0.1% Triton X-100 for 30 min, blocked in 1% BSA for 1 hour, and then incubated with CD44 PE (1:100 dilution) and CD104 eFluor 660 (1:50 dilution) antibodies at 4°C overnight. Slides were visualized using a Zeiss LSM780 confocal laser scanning system.

### Animal studies

Male BALB/C-nu/nu mice (4–6 weeks old) were purchased from the Laboratory Animal Center of Sun Yat-Sen University. For analyzing the effects of LMP1 and LMP2A on xenograft tumor growth, 2x10^5^ cells per injection (1:8 matrigel dilution) were inoculated subcutaneously into the left flanks of mice. For determining the tumor initiating cell frequencies of E, E/M and xM subpopulation, E, E/M and xM cells were counted (1:1 matrigel dilution) and inoculated subcutaneously into the left flanks of mice. Tumor initiating cell frequencies were calculated by using the extreme limiting dilution analysis (ELDA) [47].

### Histological analysis

Tissue processing, Hematoxylin and eosin (H&E) staining, and Immunohistochemistry (IHC) staining were performed as described previously [48]. Briefly, the paraffin-embedded sections of xenograft tumors were deparaffinized and rehydrated, and then subjected to high-temperature antigen retrieval. Samples were incubated with primary antibodies against Ki67 (1:100 dilution), LMP1 (1:50 dilution), LMP2A (1:50 dilution), CD34 (1:100 dilution), and ZEB1 (1:100 dilution) at 4°C overnight, followed with goat anti-rabbit HRP secondary antibody (Maxim, Cat#DAB-1031). Sections were treated with metal enhanced DAB colorimetric detection, and counterstained with hematoxylin. For detection of VM structures, PAS staining was performed using PAS staining kit (Servicebio, Cat#G1008) per manufacture’s protocol. Staining was repeated at least twice in sequential sections. Images were acquired using a Zeiss observer Z1 microscope.

### *In vitro* tube formation assay

Forty-eight-well plates were coated with Matrigel (1:1 dilute with 1640 without FBS, 100 μl/well) and incubated at 37°C for 1h to allow gelation to occur. E, E/M and xM cells were suspended in 200 μl 1640 basic medium and added on the top of the gel. The plates were incubated at 37°C with 5% CO_2_ for 6 hours and images of tube formation were captured using a ZEISS microscope. The quantification of the tube was using the software ImageJ to measure the total length of the tube from three randomly chosen fields.

### siRNA-mediated gene silencing

The siRNAs targeting LMP1 and LMP2A, as well as siCTR were transfected into E/M, xM, S26-LMP1, S26-LMP2A cells using Lipofectamine 3000 Transfection Reagent. After 72 hours, cells were harvested and subjected to flow cytometry and Western blot assays. The siRNAs were synthesis by Ribobio, China, and the sequences are as follows. siLMP1-1: TCCTACTGATGATCACCCT; siLMP1-2: GCTTACTTGTCTTAGGTAT; siLMP2A-1:GGAACGTGAATCTAATGAA; siLMP2A-2:CATATCGCAACACTGTATA.

### Statistical analysis

The two-tailed Student’s unpaired t-test was applied to compare the data from two study groups. A P value <0.05 (* denotes a P value<0.05, show 4 significant digits) was used to determine statistical significance. Error bars represent the SD (Standard Deviation) of the sample data. Data was analyzed by GraphPad Prism.

## Supporting information

S1 Fig Effects of LMP1 and LMP2A expression on the generation E-M states in HK-1 cells. **(A)** The changes of CD44 and CD104 profiles of HK-1 cells in response to LMP1 and LMP2A expression revealed by flow cytometry analysis. **(B)** The expression level of LMP1 and LMP2A were determined by Western blot. β-actin is included as a loading control.
(TIF)

S2 Fig The expression levels of HPV-18 E6/E7 and P53 genes in different NPC cell lines. Relative mRNA expression level vs. GAPDH of HPV-18 E6/E7 and P53 in CNE2, S18, S26, HK-1, and EBV-positive HK1 cells (Mean +/- SD of three biological replicates).
(TIF)

S3 Fig Effects of silencing LMP1 and LMP2A expression on plasticity of E/M and xM cells. **(A)** Purified E/M cells were transfected with siRNAs to knock down the expression of LMP1 or LMP2A. The knockdown efficiencies of these siRNAs in E/M cells were analyzed by Western blot. **(B)** The changes of CD44 and CD104 profile in E/M cells were analyzed by flow cytometry analysis. **(C)** Purified xM cells were transfected with siRNAs to knock down the expression of LMP1 or LMP2A. The knockdown efficiencies of these siRNAs in xM cells were analyzed by Western blot. **(D)** The changes of CD44 and CD104 profile in xM cells were analyzed by flow cytometry analysis.
(TIF)

## References

[1] Pattabiraman DR, Weinberg RA. Tackling the cancer stem cells—what challenges do they pose? *Nat Rev Drug Discov*. 2014;13:497–512. doi: 10.1038/nrd4253 24981363 PMC4234172

[2] Wilson MM, Weinberg RA, Lees JA, Guen VJ. Emerging Mechanisms by which EMT Programs Control Stemness. *Trends Cancer*. 2020;6:775–80. doi: 10.1016/j.trecan.2020.03.011 32312682

[3] Pastushenko I, Brisebarre A, Sifrim A, Fioramonti M, Revenco T, Boumahdi S, et al. Identification of the tumour transition states occurring during EMT. *Nature*. 2018;556:463–8. doi: 10.1038/s41586-018-0040-3 29670281

[4] Grigore AD, Jolly MK, Jia D, Farach-Carson MC, Levine H. Tumor Budding: The Name is EMT. Partial EMT. *J Clin Med*. 2016;5:51 doi: 10.3390/jcm5050051 27136592 PMC4882480

[5] Jordan NV, Johnson GL, Abell AN. Tracking the intermediate stages of epithelial-mesenchymal transition in epithelial stem cells and cancer. *Cell Cycle*. 2011;10:2865–73. doi: 10.4161/cc.10.17.17188 21862874 PMC3218599

[6] Nieto MA, Huang RY, Jackson RA, Thiery JP. EMT: 2016. *Cell*. 2016;166:21–45. doi: 10.1016/j.cell.2016.06.028 27368099

[7] Kroger C, Afeyan A, Mraz J, Eaton EN, Reinhardt F, Khodor YL, et al. Acquisition of a hybrid E/M state is essential for tumorigenicity of basal breast cancer cells. *Proc Natl Acad Sci USA*. 2019;116:7353–62. doi: 10.1073/pnas.1812876116 30910979 PMC6462070

[8] Lo KW, To KF, Huang DP. Focus on nasopharyngeal carcinoma. *Cancer Cell*. 2004;5:423–8. doi: 10.1016/s1535-6108(04)00119-9 15144950

[9] Young LS, Dawson CW. Epstein-Barr virus and nasopharyngeal carcinoma. *Chin J Cancer*. 2014;33:581–90. doi: 10.5732/cjc.014.10197 25418193 PMC4308653

[10] Wang J, Guo LP, Chen LZ, Zeng YX, Lu SH. Identification of cancer stem cell-like side population cells in human nasopharyngeal carcinoma cell line. *Cancer Res*. 2007;67:3716–24. doi: 10.1158/0008-5472.CAN-06-4343 17440084

[11] Su J, Xu XH, Huang Q, Lu MQ, Li DJ, Xue F, et al. Identification of cancer stem-like CD44+ cells in human nasopharyngeal carcinoma cell line. *Arch Med Res*. 2011;42:15–21. doi: 10.1016/j.arcmed.2011.01.007 21376257

[12] Lun SW, Cheung ST, Cheung PF, To KF, Woo JK, Choy KW, et al. CD44+ cancer stem-like cells in EBV-associated nasopharyngeal carcinoma. *PloS One*. 2012;7:e52426. doi: 10.1371/journal.pone.0052426 23285037 PMC3528656

[13] Brooks L, Yao QY, Rickinson AB, Young LS. Epstein-Barr virus latent gene transcription in nasopharyngeal carcinoma cells: coexpression of EBNA1, LMP1, and LMP2 transcripts. *J Virol*. 1992;66:2689–97. doi: 10.1128/JVI.66.5.2689-2697.1992 1313894 PMC241023

[14] Yoshizaki T. Promotion of metastasis in nasopharyngeal carcinoma by Epstein-Barr virus latent membrane protein-1. *Histol Histopathol*. 2002;17:845–50. doi: 10.14670/HH-17.845 12168795

[15] Horikawa T, Yang J, Kondo S, Yoshizaki T, Joab I, Furukawa M, et al. Twist and Epithelial-Mesenchymal Transition Are Induced by the EBV Oncoprotein Latent Membrane Protein 1 and Are Associated with Metastatic Nasopharyngeal Carcinoma. *Cancer Res*. 2007;67:1970. doi: 10.1158/0008-5472.CAN-06-3933 17332324

[16] Horikawa T, Yoshizaki T, Kondo S, Furukawa M, Kaizaki Y, Pagano JS. Epstein-Barr Virus latent membrane protein 1 induces Snail and epithelial-mesenchymal transition in metastatic nasopharyngeal carcinoma. *Br J Cancer*. 2011;104:1160–7. doi: 10.1038/bjc.2011.38 21386845 PMC3068490

[17] Shair KHY, Schnegg CI, Raab-Traub N. Epstein-Barr virus latent membrane protein-1 effects on junctional plakoglobin and induction of a cadherin switch. *Cancer Res*. 2009;69:5734–42. doi: 10.1158/0008-5472.CAN-09-0468 19584275 PMC2771661

[18] Kong QL, Hu LJ, Cao JY, Huang YJ, Xu LH, Liang Y, et al. Epstein-Barr virus-encoded LMP2A induces an epithelial-mesenchymal transition and increases the number of side population stem-like cancer cells in nasopharyngeal carcinoma. *PLoS Pathog*. 2010;6:e1000940. doi: 10.1371/journal.ppat.1000940 20532215 PMC2880580

[19] Qian CN, Berghuis B, Tsarfaty G, Bruch M, Kort EJ, Ditlev J, et al. Preparing the “Soil”: The Primary Tumor Induces Vasculature Reorganization in the Sentinel Lymph Node before the Arrival of Metastatic Cancer Cells. *Cancer Res*. 2006;66:10365–76. doi: 10.1158/0008-5472.CAN-06-2977 17062557

[20] Thiery JP, Acloque H, Huang RY, Nieto MA. Epithelial-mesenchymal transitions in development and disease. *Cell*. 2009;139:871–90. doi: 10.1016/j.cell.2009.11.007 19945376

[21] Lamouille S, Xu J, Derynck R. Molecular mechanisms of epithelial-mesenchymal transition. *Nat Rev Mol Cell Biol*. 2014;15:178–96. doi: 10.1038/nrm3758 24556840 PMC4240281

[22] Krebs AM, Mitschke J, Lasierra Losada M, Schmalhofer O, Boerries M, Busch H, et al. The EMT-activator Zeb1 is a key factor for cell plasticity and promotes metastasis in pancreatic cancer. *Nat Cell Biol*. 2017;19:518–29. doi: 10.1038/ncb3513 28414315

[23] Huang DP, Ho JH, Poon YF, Chew EC, Saw D, Lui M, et al. Establishment of a cell line (NPC/HK1) from a differentiated squamous carcinoma of the nasopharynx. *Int J Cancer*. 1980;26:127–32. doi: 10.1002/ijc.2910260202 6259064

[24] Folberg R, Hendrix MJ, Maniotis AJ. Vasculogenic mimicry and tumor angiogenesis. *Am J Pathol*. 2000;156:361–81. doi: 10.1016/S0002-9440(10)64739-6 10666364 PMC1850026

[25] Zhang S, Zhang D, Sun B. Vasculogenic mimicry: current status and future prospects. *Cancer Lett*. 2007;254:157–64. doi: 10.1016/j.canlet.2006.12.036 17306454

[26] Li B, Mao X, Wang H, Su G, Mo C, Cao K, et al. Vasculogenic mimicry in bladder cancer and its association with the aberrant expression of ZEB1. Onco Lett. 2018;15:5193–200. doi: 10.3892/ol.2018.7975 29552157 PMC5840607

[27] Chen YS, Chen ZP. Vasculogenic mimicry: a novel target for glioma therapy. *Chin J Cancer*. 2014;33:74–9. doi: 10.5732/cjc.012.10292 23816560 PMC3935008

[28] Xiang T, Lin YX, Ma W, Zhang HJ, Chen KM, He GP, et al. Vasculogenic mimicry formation in EBV-associated epithelial malignancies. *Nat Commun*. 2018;9:5009. doi: 10.1038/s41467-018-07308-5 30479336 PMC6258759

[29] Xu S, Bai J, Zhuan Z, Li B, Zhang Z, Wu X, et al. EBV-LMP1 is involved in vasculogenic mimicry formation via VEGFA/VEGFR1 signaling in nasopharyngeal carcinoma. *Oncol Rep*. 2018;40:377–84. doi: 10.3892/or.2018.6414 29749553

[30] Farrell PJ. Epstein-Barr virus immortalizing genes. *Trends Microbiol*. 1995;3:105–9. doi: 10.1016/s0966-842x(00)88891-5 7773587

[31] Wang D, Liebowitz D, Kieff E. An EBV membrane protein expressed in immortalized lymphocytes transforms established rodent cells. *Cell*. 1985;43:831–40. doi: 10.1016/0092-8674(85)90256-9 3000618

[32] Baichwal VR, Sugden B. Transformation of Balb 3T3 cells by the BNLF-1 gene of Epstein-Barr virus. *Oncogene*. 1988;2:461–7. 2836780

[33] Tsao SW, Wang X, Liu Y, Cheung YC, Feng H, Zheng Z, et al. Establishment of two immortalized nasopharyngeal epithelial cell lines using SV40 large T and HPV16E6/E7 viral oncogenes. *Biochim biophys Acta*. 2002;1590:150–8. doi: 10.1016/s0167-4889(02)00208-2 12063178

[34] Raab-Traub N. Epstein-Barr virus in the pathogenesis of NPC. *Semin Cancer Biol*. 2002;12:431–41. doi: 10.1016/s1044579x0200086x 12450729

[35] Dawson CW, Port RJ, Young LS. The role of the EBV-encoded latent membrane proteins LMP1 and LMP2 in the pathogenesis of nasopharyngeal carcinoma (NPC). *Semin Cancer Biol*. 2012;22:144–53. doi: 10.1016/j.semcancer.2012.01.004 22249143

[36] Niedobitek G, Fahraeus R, Herbst H, Latza U, Ferszt A, Klein G, et al. The Epstein-Barr virus encoded membrane protein (LMP) induces phenotypic changes in epithelial cells. *Virchows Arch B Cell Pathol Incl Mol Pathol*. 1992;62:55–9. doi: 10.1007/BF02899665 1352076

[37] Morris MA, Young LS, Dawson CW. DNA tumour viruses promote tumour cell invasion and metastasis by deregulating the normal processes of cell adhesion and motility. *Eur J Cell Biol*. 2008;87:677–97. doi: 10.1016/j.ejcb.2008.03.005 18468721

[38] Kondo S, Wakisaka N, Muramatsu M, Zen Y, Endo K, Murono S, et al. Epstein-Barr virus latent membrane protein 1 induces cancer stem/progenitor-like cells in nasopharyngeal epithelial cell lines. *J Virol*. 2011;85:11255–64. doi: 10.1128/JVI.00188-11 21849440 PMC3194961

[39] Yang CF, Yang GD, Huang TJ, Li R, Chu QQ, Xu L, et al. EB-virus latent membrane protein 1 potentiates the stemness of nasopharyngeal carcinoma via preferential activation of PI3K/AKT pathway by a positive feedback loop. *Oncogene*. 2016;35:3419–31. doi: 10.1038/onc.2015.402 26568302

[40] Lam N, Sugden B. CD40 and its viral mimic, LMP1: similar means to different ends. *Cell Signal*. 2003;15:9–16. doi: 10.1016/s0898-6568(02)00083-9 12401515

[41] Péguet-Navarro J, Dalbiez-Gauthier C, Moulon C, Berthier O, Réano A, Gaucherand M, et al. CD40 ligation of human keratinocytes inhibits their proliferation and induces their differentiation. *J Immunol*. 1997;158:144–52. 8977185

[42] Young LS, Eliopoulos AG, Gallagher NJ, Dawson CW. CD40 and epithelial cells: across the great divide. *Immunol Today*. 1998;19:502–6. doi: 10.1016/s0167-5699(98)01340-1 9818543

[43] Deregibus MC, Buttiglieri S, Russo S, Bussolati B, Camussi G. CD40-dependent activation of phosphatidylinositol 3-kinase/Akt pathway mediates endothelial cell survival and in vitro angiogenesis. *J Biol Chem*. 2003;278:18008–14. doi: 10.1074/jbc.M300711200 12637493

[44] Zhu N, Wang Q, Wu Z, Wang Y, Zeng M, Yuan Y. Role of mTOR Complexes in the Maintenance of Nasopharyngeal Carcinoma Cancer Stem Cells. *J Virol*. (revision)

[45] Visvader JE, Lindeman GJ. Cancer stem cells: current status and evolving complexities. *Cell Stem Cell*. 2012;10:717–28. doi: 10.1016/j.stem.2012.05.007 22704512

[46] Dagogo-Jack I, Shaw AT. Tumour heterogeneity and resistance to cancer therapies. *Nat Rev Clin oncol*. 2018;15:81–94. doi: 10.1038/nrclinonc.2017.166 29115304

[47] Hu Y, Smyth GK. ELDA: extreme limiting dilution analysis for comparing depleted and enriched populations in stem cell and other assays. *J Immunol Methods*. 2009;347:70–8. doi: 10.1016/j.jim.2009.06.008 19567251

[48] Li Y, Zhong C, Liu D, Yu W, Chen W, Wang Y, et al. Evidence for Kaposi Sarcoma Originating from Mesenchymal Stem Cell through KSHV-induced Mesenchymal-to-Endothelial Transition. *Cancer Res*. 2018;78:230–45. doi: 10.1158/0008-5472.CAN-17-1961 29066510 PMC5754241

