# Supplementary figures and images for: EBV latent membrane proteins promote hybrid epithelial-mesenchymal and extreme mesenchymal states of nasopharyngeal carcinoma cells for tumorigenicity
Source: PLoS Pathog. 2021 Aug 18;17(8):e1009873. doi: 10.1371/journal.ppat.1009873 (PMC8405006; doi:10.1371/journal.ppat.1009873)

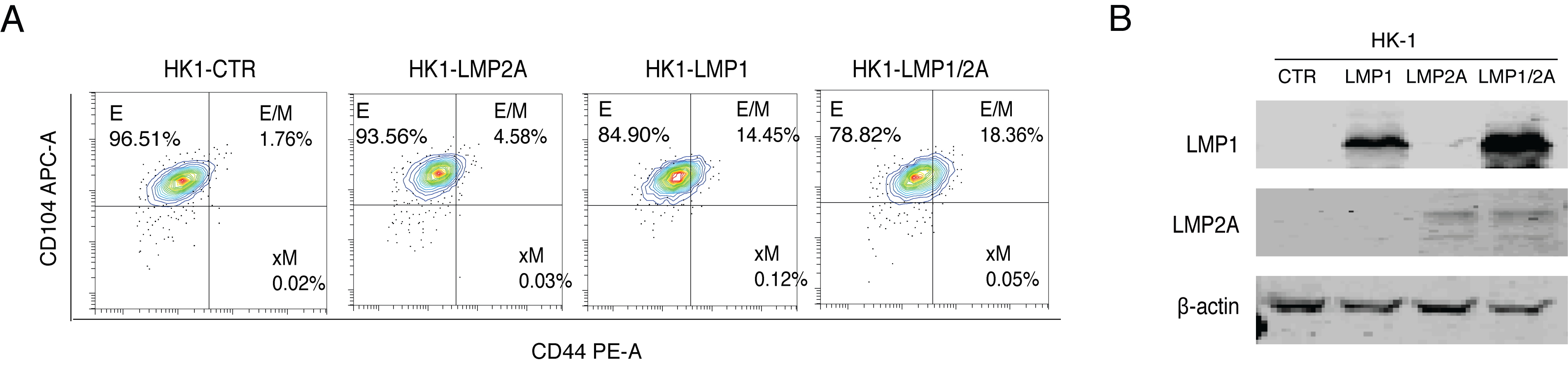

Supplement: S1 Fig — (A) The changes of CD44 and CD104 profiles of HK-1 cells in response to LMP1 and LMP2A expression revealed by flow cytometry analysis. (B) The expression level of LMP1 and LMP2A were determined by Western blot. β-actin is included as a loading control. (TIF) [file ppat.1009873.s001.tif]

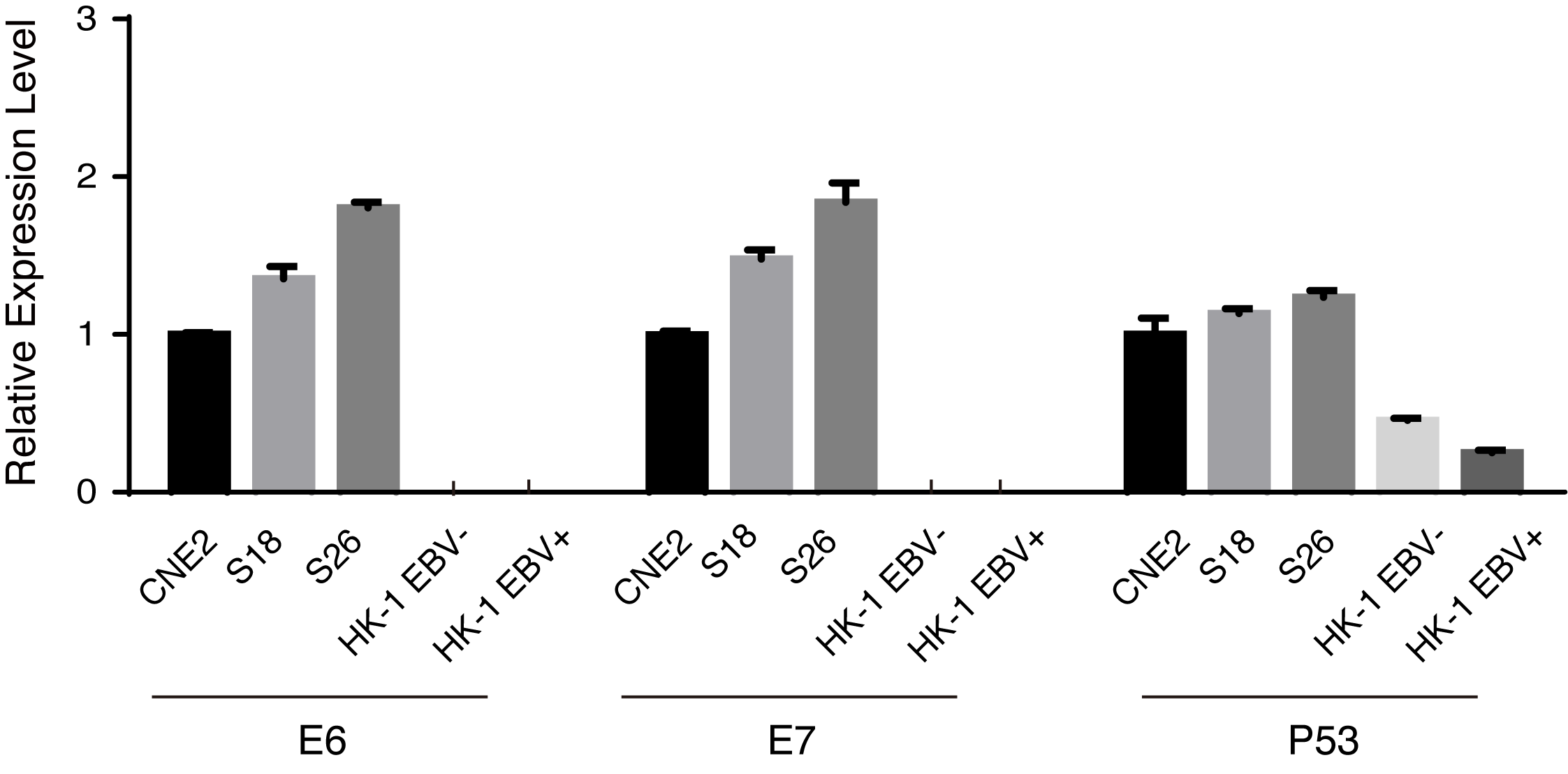

Supplement: S2 Fig — Relative mRNA expression level vs. GAPDH of HPV-18 E6/E7 and P53 in CNE2, S18, S26, HK-1, and EBV-positive HK1 cells (Mean +/- SD of three biological replicates). (TIF) [file ppat.1009873.s002.tif]

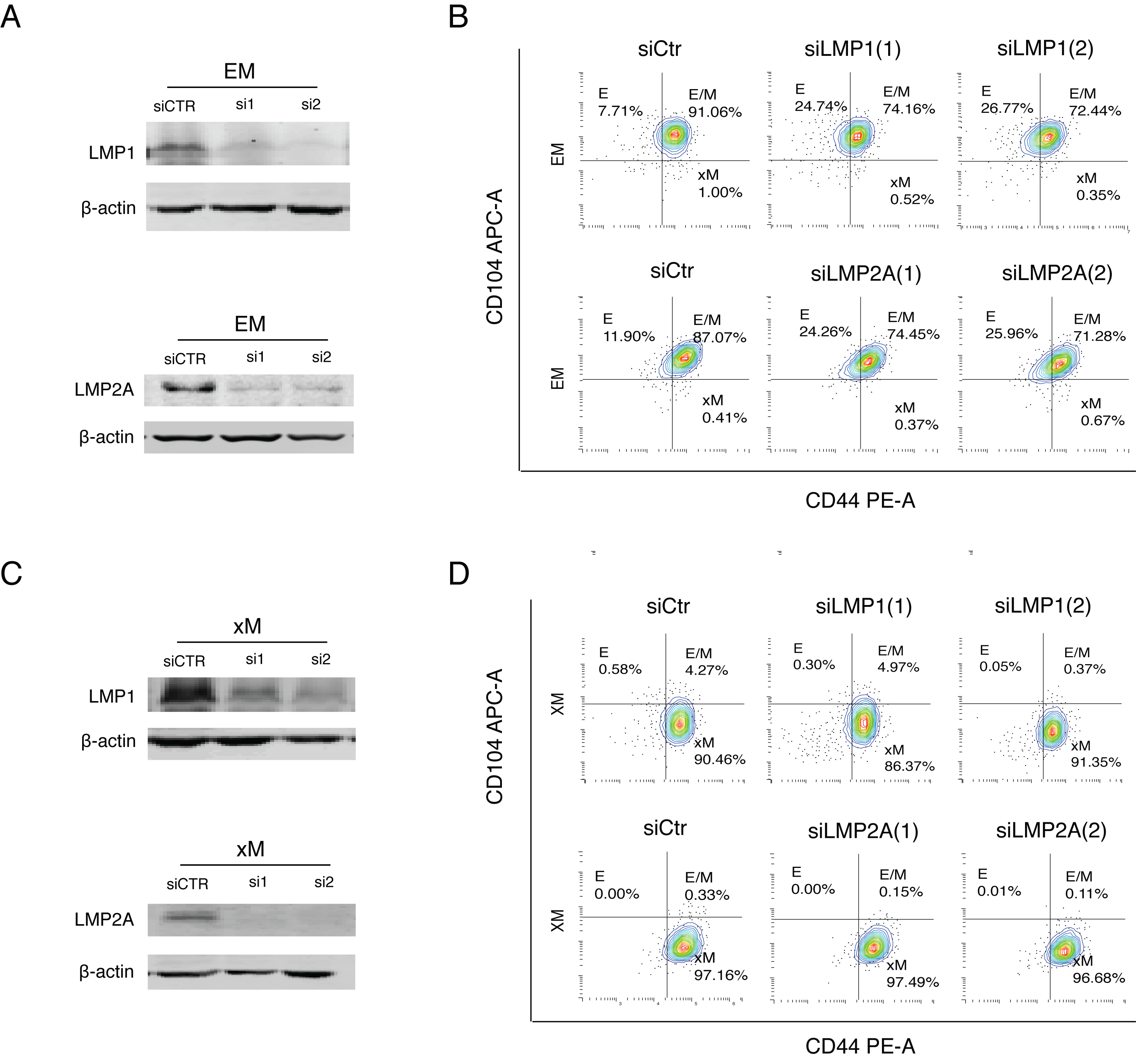

Supplement: S3 Fig — (A) Purified E/M cells were transfected with siRNAs to knock down the expression of LMP1 or LMP2A. The knockdown efficiencies of these siRNAs in E/M cells were analyzed by Western blot. (B) The changes of CD44 and CD104 profile in E/M cells were analyzed by flow cytometry analysis. (C) Purified xM cells were transfected with siRNAs to knock down the expression of LMP1 or LMP2A. The knockdown efficiencies of these siRNAs in xM cells were analyzed by Western blot. (D) The changes of CD44 and CD104 profile in xM cells were analyzed by flow cytometry analysis. (TIF) [file ppat.1009873.s003.tif]
